# Novel Antimicrobial Agents: Fluorinated 2-(3-(Benzofuran-2-yl) pyrazol-1-yl)thiazoles

**DOI:** 10.1155/2013/986536

**Published:** 2013-09-11

**Authors:** Hanan A. Mohamed, Ehab Abdel-Latif, Bakr F. Abdel-Wahab, Ghada E. A. Awad

**Affiliations:** ^1^Applied Organic Chemistry Department, National Research Centre, Dokki, Giza 12622, Egypt; ^2^Department of Chemistry, Faculty of Science, Mansoura University, Mansoura 35516, Egypt; ^3^Chemistry of Natural and Microbial Products, National Research Center, Dokki, Giza 12622, Egypt

## Abstract

A new series of 2-pyrazolin-1-ylthiazoles **8a–d** and **13–16** was synthesized by cyclization of *N*-thiocarboxamide-2-pyrazoline with different haloketones and 2,3-dichloroquinoxaline. The structures of the new compounds were confirmed by elemental analyses as well as NMR, IR, and mass spectral data. The newly synthesized compounds were evaluated for their antimicrobial activities, and also their minimum inhibitory concentration (MIC) against most of test organisms was performed. Amongst the tested ones, compound **8c** displayed excellent antimicrobial activity.

## 1. Introduction

Pyrazolines are nitrogen-containing heterocyclic compounds, well known for their pronounced biological activity. These biological activities include antibacterial [1], antifungal [2], herbicidal [3], and insecticidal activities [4]. It was demonstrated that the combination of pyrazole with azole ring, linked to each by one sigma bond, led to more biologically active targets; for example, pyrazolylthiazoles showed excellent antimicrobial activities [5]. Continuing our work in this research field [6–9] and in an attempt to identify new and potent antimicrobial agents, we tried here to generate new benzofuryl 2-pyrazolin-1-ylthiazoles as antimicrobial agents using simple methods.

## 2. Results and Discussion

### 2.1. Chemistry

The starting pyrazoline-1-carbothioamide **5** was prepared by treatment of 2-acetylbenzofuran **1** with equivalent of 4-flurobenzaldehyde **2** in the presence of 10% alcoholic NaOH in 90% ethanol with stirring at room temperature to give chaconne **3**. Reaction of chalcone **3** with equivalent amount of thiosemicarbazide was performed in ethanol in the presence of 2.5 equivalent of sodium hydroxide to the target precursor **5**.

**Scheme 1:**
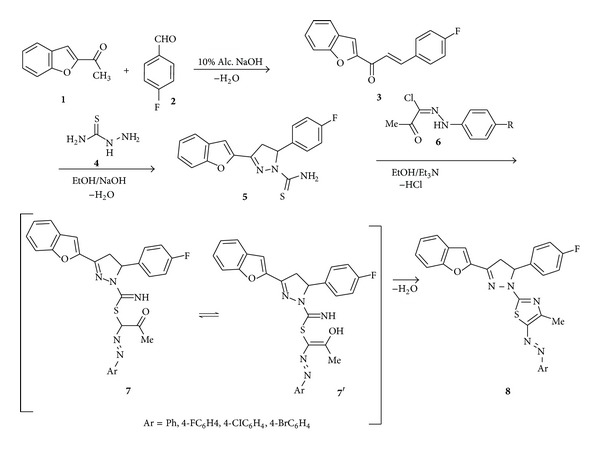


The resulting pyrazoline-1-carbothioamide **5** was cyclized to the corresponding 2-(3-(benzofuran-2-yl)-5-(4-fluorophenyl)-4,5-dihydro-1*H*-pyrazol-1-yl)-4-methyl-5-(*p*-subs.phenyldiazenyl)thiazole derivatives **8a**–**d** by reaction with hydrazonoyl halides **6a**–**d** in anhydrous ethanol and in the presence of an equivalent of triethylamine (Scheme 1).

The reaction product structures were elucidated by means of NMR, MS spectroscopy, and elemental analyses (Table 1).

For example, ^1^H NMR spectra of **8a**–**d** contained two doublet-doublet and one triplet signals due to the presence of CH_2_ adjacent asymmetric carbon. The mass spectra of **8a**–**d** showed the molecular ion peaks at *m/z* 481, 499, 515 and 561, respectively in agreement with the calculated masses.

**Scheme 2:**
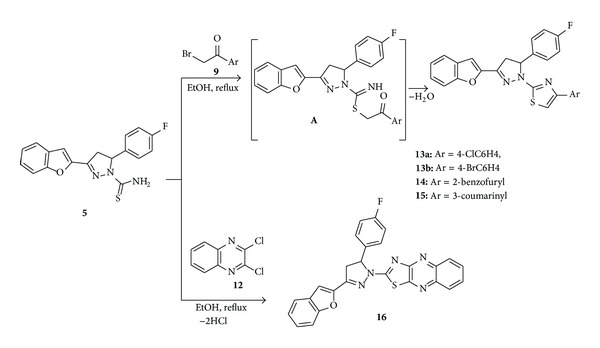


The reaction between pyrazoline-1-carbothioamide **5** and the equivalent amount of *α*-haloketones, for example, phenacyl bromides derivatives, 2-bromoacetylbenzofuran, and 3-bromoacetylcoumarin, was performed in refluxing ethanol to yield pyrazol-1-ylthiazoles **13**–**15** in good yields *via* the intermediates **A** (Scheme 2). Also, pyrazoline-1-carbothioamide **5** reacted with 2,3-dichloroquinoxaline to give pyrazolin-1-ylthiazolo[5,4-b]quinoxaline **16** in moderate yield.

The ^1^H NMR of compounds **13**–**16** indicate the disappearance of NH_2_ signal due to blocking of NH_2_ with thiazole nucleus. The mass spectra of **13a,b**–**16** showed the molecular ion peaks at *m/z* 473, 518, 479, 507, and 465, respectively in agreement with the calculated masses.

### 2.2. Biological Activity

#### 2.2.1. Antimicrobial Activity

All the new synthesized compounds were screened for their antibacterial and antifungal activities at 100 *μ*g/mL concentration against four *Gram-positive bacteria* (*Staphylococcus aureus* ATCC 29213; *B. subtilis ATCC6633*; *B. megaterium ATCC *9885; *Sarcina lutea*), three *Gram-negative bacteria* (*Klebsiella pneumoniae* ATCC13883; *Pseudomonas aeruginosa* ATCC27953; *E. coli ATCC 25922*), and two yeast (*Saccharomyces cerevisiae* and *Candida albicans* NRRL Y-477). Ciprofloxacin and ketoconazole were respectively used as standard antibacterial and antifungal references, respectively. Most of the newly synthesized compounds showed good antimicrobial activities with respect to the control drugs. The results of antimicrobial activities were shown in Table 2. Data showed that most of compounds have superior significant antifungal potency to antibacterial potency. Compound **8c** exhibited the highest potency against all tested organisms with respect to reference drugs. Compounds **8d** and **13b** inhibited the growth of *Staphylococcus aureus *ATCC 29213 with inhibition zones 23, 22 mm, respectively, while compound **5** showed excellent activities against* Klebsiella pneumoniae* ATCC13883; *Pseudomonas aeruginosa* ATCC27953; and *E. coli ATCC 25922* with inhibition zone about 24 mm. Also, compound **8c** showed the highest activity against *Staphylococcus aureus* ATCC 29213, *Saccharomyces cerevisiae*, and *Candida albicans* NRRL Y-477 with inhibition zone about 23 mm.

#### 2.2.2. Minimum Inhibitory Concentration (MIC)

The minimum inhibitory concentration (MIC) of the synthesized compounds against highly inhibited organisms is reported in Table 3. Compounds **5** revealed low MIC (200 *μ*g/mL) against *Staphylococcus aureus* ATCC 29213, *B. megaterium ATCC *9885, and *Candida albicans* NRRL Y-477, respectively. On the other hand, compound **8a** exhibited high MIC (16 *μ*g/mL) against *B. subtilis ATCC6633* (Table 3).

## 3. Experimental

### 3.1. Chemistry

All melting points were taken on Electrothermal IA 9000 series digital melting point apparatus. Elemental analytical data were carried from the microanalytical unit, Cairo University, Giza, Egypt. The IR spectra were recorded in potassium bromide disks on a JASCO FT/IR-6100. ^1^H NMR spectra were run on JOEL-ECA 500 MHz in deuterateddimethyl sulphoxide (DMSO-d_6_). Chemical shifts values (*δ*) are given in parts per million (ppm). The mass spectra were performed using mass Varian MAT CH-5 spectrometer at 70 eV. (*E*)-1-(benzofuran-2-yl)-3-(4-fluorophenyl)prop-2-en-1-one **3** [10]; hydrazonoyl halides [11]; 1-(benzofuran-2-yl)-2-bromoethanone **9** [12]; 3-(2-bromoacetyl)-2H-chromen-2-one **10** [13]; and 2,3-dichloroquinoxaline [14] were prepared according to the literature.

#### 3.1.1. 3-(Benzofuran-2-yl)-5-(4-fluorophenyl)-4,5-dihydro-1*H*-pyrazole-1-carbothioamide (**5**)

To a suspension of chalcone **3** (10 mmol, 2.66 g) and sodium hydroxide (25 mmol, 1.0 g) in ethanol (50 mL), thiosemicarbazide (12 mmol, 1.1 g) was added. The mixture was refluxed for 12 h, then left to cool; the solid product was filtered off, washed with ethanol, and dried.

Yield 52%; m.p. 260-2°C; IR (KBr) *ν*max/cm^−1^ 3460, 3335 (NH_2_); ^1^H NMR (DMSO-d_6_) *δ* 3.09, 3.14 (dd, 1H, CH, *J* = 3.05 Hz, *J* = 3.05 Hz), 3.94, 4.06 (dd, 1H, CH, *J* = 11.45 Hz, *J* = 11.5 Hz), 5.88 (t, 1H, CH, *J* = 3.05 Hz, *J* = 7.65 Hz), 7.13–7.43 (m, 9H, Ar-H), 9.44 (s, 2H, NH_2_, D_2_O-exchangeable); MS *m/z* (%): 339 (M^+^, 75), 60 (100).

#### 3.1.2. General Procedure for Compounds **8a–d**; **13a–d**; **14**; **15;** and **16**

To a suspension of compound **5** (1 mmol, 0.34 g) in ethanol (20 mL), the 1 mmol of appropriate reagent {(appropriate hydrozonoyl chlorides **6** + Et_3_N); (appropriate phenacyl bromides **9**); (2-bromoacetylbenzofuran **10**); (3-bromoacetylcoumarin **11**); or (3,4-dichloroquinoxaline **12**)} was added and heated under reflux for 4 h. After cooling, the precipitate was collected by suction filtration.

#### 3.1.3. 2-(3-(Benzofuran-2-yl)-5-(4-fluorophenyl)-4,5-dihydro-1*H*-pyrazol-1-yl)-4-methyl-5-(phenyldiazenyl)thiazole (**8a**)

Yield 58%; m.p.180-2°C; ^1^H NMR (DMSO-d_6_) *δ* 2.54 (s, 3H, CH_3_), 4.09, 4.11 (2dd, 2H, CH, *J* = 10.7 Hz, *J* = 9.95 Hz), 5.87 (t, 1H, CH, *J* = 10.7 Hz, *J* = 9.95 Hz), 7.18–7.73 (m, 14H, Ar-H); MS *m/z* (%): 481 (M^+^, 75), 95 (100).

#### 3.1.4. 2-(3-(Benzofuran-2-yl)-5-(4-fluorophenyl)-4,5-dihydro-1*H*-pyrazol-1-yl)-5-((4-fluorophenyl)diazenyl)-4-methylthiazole (**8b**)

Yield 66%; m.p. 201-3°C; ^1^H NMR (DMSO-d_6_) *δ* 2.54 (s, 3H, CH_3_), 4.07, 4.12 (2dd, 2H, CH, *J* = 10.7 Hz, *J* = 9.95 Hz), 5.89 (t, 1H, CH, *J* = 10.7 Hz, *J* = 9.95 Hz), 7.19–7.73 (m, 13H, Ar-H); MS *m/z* (%): 499 (M^+^, 80), 95 (100).

#### 3.1.5. 2-(3-(Benzofuran-2-yl)-5-(4-fluorophenyl)-4,5-dihydro-1*H*-pyrazol-1-yl)-5-((4-chlorophenyl)diazenyl)-4-methylthiazole (**8c**)

Yield 72%; m.p. 206-8°C; ^1^H NMR (DMSO-d_6_) *δ* 2.53 (s, 3H, CH_3_), 4.10, 4.34 (2dd, 2H, CH, *J* = 10.7 Hz, *J* = 9.95 Hz), 5.89 (t, 1H, CH, *J* = 10.7 Hz, *J* = 9.95 Hz), 7.19–7.73 (m, 13H, Ar-H); MS *m/z* (%): 515 (M^+^, 70), 95 (100).

#### 3.1.6. 2-(3-(Benzofuran-2-yl)-5-(4-fluorophenyl)-4,5-dihydro-1*H*-pyrazol-1-yl)-5-((4-bromophenyl)diazenyl)-4-methylthiazole (**8d**)

Yield 75%; m.p. 128-30°C; ^1^H NMR (DMSO-d_6_) *δ* 2.55 (s, 3H, CH_3_), 4.10, 4.34 (2dd, 2H, CH, *J* = 10.7 Hz, *J* = 9.95 Hz), 5.87 (t, 1H, CH, *J* = 10.7 Hz, *J* = 9.95 Hz), 7.19–7.72 (m, 13H, Ar-H); MS *m/z* (%): 561 (M^+^, 62), 95 (100).

#### 3.1.7. 2-(3-(Benzofuran-2-yl)-5-(4-fluorophenyl)-4,5-dihydro-1*H*-pyrazol-1-yl)-4-(4-chlorophenyl)thiazole (**13a**)

Yield 49%; m.p. 176-8°C; ^1^H NMR (DMSO-d_6_) *δ* 4.01–4.05 (dd, 2H, CH_2_, *J* = 11.45 Hz, *J* = 10.33 Hz), 5.73 (t, 1H, CH, *J* = 5.35 Hz, *J* = 6.1 Hz), 7.17–7.92 (m, 14H, Ar-H); MS *m/z* (%): 473 (M^+^, 80), 91 (100).

#### 3.1.8. 2-(3-(Benzofuran-2-yl)-5-(4-fluorophenyl)-4,5-dihydro-1*H*-pyrazol-1-yl)-4-(4-bromophenyl)thiazole (**13b**)

Yield 58%; m.p. 208-10°C; ^1^H NMR (DMSO-d_6_) *δ* 4.01–4.08 (dd, 2H, CH_2_, *J* = 11.45 Hz, *J* = 10.33 Hz), 5.71 (t, 1H, CH, *J* = 5.35 Hz, *J* = 6.1 Hz), 7.17–7.92 (m, 14H, Ar-H); MS *m/z* (%): 518 (M^+^, 49), 91 (100).

#### 3.1.9. 4-(Benzofuran-2-yl)-2-(3-(benzofuran-2-yl)-5-(4-fluorophenyl)-4,5-dihydro-1*H*-pyrazol-1-yl)thiazole (**14**)

Yield 63%; m.p. 252-4°C; ^1^H NMR (DMSO-d_6_) *δ* 4.04–4.07 (dd, 2H, CH_2_, *J* = 11.45 Hz, *J* = 10.33 Hz), 5.73 (t, 1H, CH, *J* = 5.35 Hz, *J* = 6.1 Hz), 6.88 (s, 2H, benzofuryl-H), 7.19–7.70 (m, 13H, Ar-H); MS *m/z* (%): 479 (M^+^, 100).

#### 3.1.10. 3-(2-(3-(Benzofuran-2-yl)-5-(4-fluorophenyl)-4,5-dihydro-1*H*-pyrazol-1-yl)thiazol-4-yl)-2*H*-chromen-2-one (**15**)

Yield 74%; m.p. 233-4°C; ^1^H NMR (DMSO-d_6_) *δ* 4.05, 4.08 (dd, 2H, CH_2_, *J* = 11.45 Hz, *J* = 10.33 Hz), 5.72 (t, 1H, CH, *J* = 5.35 Hz, *J* = 6.1 Hz), 7.22–7.72 (m, 14H, Ar-H), 8.23 (s, 1H, coumarinyl-H); MS *m/z* (%): 507 (M^+^, 100).

#### 3.1.11. 2-(3-(Benzofuran-2-yl)-5-(4-fluorophenyl)-4,5-dihydro-1*H*-pyrazol-1-yl)thiazolo[5,4-*b*]quinoxaline (**16**)

Yield 74%; m.p. 256-8°C; ^1^H NMR (DMSO-d_6_) *δ* 4.02, 4.06 (dd, 2H, CH_2_, *J* = 11.45 Hz, *J* = 10.33 Hz), 5.70 (t, 1H, CH, *J* = 5.35 Hz, *J* = 6.1 Hz), 7.22–7.72 (m, 13H, Ar-H); MS *m/z* (%): 465 (M^+^, 100).

### 3.2. Antimicrobial Activity

Chemical compounds were individually tested against a panel of Gram-positive and Gram-negative bacterial pathogens and yeast. Antimicrobial tests were carried out by the agar well diffusion method [15] using 100 *μ*L of suspension containing 1 × 10^8^ CFU/mL of pathological tested bacteria and 1 × 10^6^ CFU/mL of yeast spread on nutrient agar (NA) and Sabourund dextrose agar (SDA), respectively. After the media had cooled and solidified, wells (10 mm in diameter) were made in the solidified agar and loaded with 100 *μ*L of tested compound solution prepared by dissolving 100 mg of the chemical compound in one mL of dimethyl sulfoxide (DMSO). The inculcated plates were then incubated for 24 h at 37°C for bacteria and at 28°C for yeast. Negative controls were prepared using DMSO employed for dissolving the tested compound. Ciprofloxacin (50 *μ*g/mL) and ketoconazole (50 *μ*g/mL) were used as standard for antibacterial and antifungal activities respectively. After incubation time, antimicrobial activity was evaluated by measuring the zone of inhibition against the test organisms and compared with that of the standard. The observed zone of inhibition is presented in Table 2. Antimicrobial activities were expressed as inhibition diameter zones in millimeters (mm). The experiment was carried out in triplicate, and the average zone of inhibition was calculated.

### 3.3. Minimal Inhibitory Concentration (MIC) Measurement

The bacteriostatic activity of the active compounds (having inhibition zones (IZ) ≥ 18 mm) was then evaluated using the twofold serial dilution technique [16]. Twofold serial dilutions of the tested compounds solutions were prepared using the proper nutrient broth. The final concentration of the solutions was 200, 100, 50, and 25 *μ*g/mL. The tubes were then inoculated with the test organisms, grown in their suitable broth at 37°C for 24 hours for bacteria (about 1 × 10^8^ CFU/mL and 1 × 10^6^ CFU/mL of yeast), and each 5 mL received 0.1 mL of the above inoculum and incubated at 37°C for 24 hours. The lowest concentration showing no growth was taken as the minimum inhibitory concentration (MIC).

## 4. Conclusion

Novel pyrazolylthiazoles, with potential antimicrobial activity, were prepared from available 2-acetylbenzofuran. Firstly, chalcone **3** was obtained by condensation with 4-fluorobenzaldehyde in alcoholic NaOH. This precursor reacted with thiosemicarbazide in strong basic medium to afford the *N*-pyrazoline thioamide **5**. Reaction of the latter with different haloketones and 2,3-dichloroquinoxaline gave the target pyrzolinylthiazoles **8**–**16**. The new compounds were tested for their antimicrobial activities and significant activities due to presence of three nucleuses: benzofuran, pyrazole, and thiazole. Also, some substituent increases the antimicrobial activities such as chloro substituent in compounds **8c** and **13a**.

## Figures and Tables

**Table 1:** Characteristic data of the synthesized compounds.

Entry	Mol. formula (M.Wt)	**Calcd.**		
		Found		
		C%	H%	N%
**5**	C_18_H_14_FN_3_OS (339.39)	**63.70** 64.03	**4.16** 4.20	**12.38** 12.51
**8a**	C_27_H_20_FN_5_OS (481.54)	**67.34** 67.54	**4.19** 4.31	**14.54** 14.35
**8b**	C_27_H_19_F_2_N_5_OS (499.53)	**64.92** 64.98	**3.83** 3.90	**14.02** 14.22
**8c**	C_27_H_19_ClFN_5_OS (515.99)	**62.85** 62.94	**3.71** 3.83	**13.57** 13.66
**8d**	C_27_H_19_BrFN_5_OS (560.44)	**57.86** 57.89	**3.42** 3.39	**12.50** 12.59
**13a**	C_26_H_17_ClFN_3_OS (473.95)	**65.89** 65.93	**3.62** 3.59	**8.87** 8.93
**13b**	C_26_H_17_BrFN_3_OS (518.40)	**60.24** 60.30	**3.31** 3.39	**8.11** 8.31
**14**	C_28_H_18_FN_3_O_2_S (479.52)	**70.13** 70.26	**3.78** 3.82	**8.76** 8.89
**15**	C_29_H_18_FN_3_O_3_S (507.53)	**68.63** 68.77	**3.57** 3.66	**8.28** 8.33
**16**	C_26_H_16_FN_5_OS (465.50)	**67.08** 67.14	**3.46** 3.50	**15.04** 15.11

**Table 2:** Antimicrobial activity expressed as inhibition diameter zones in millimeters (mm) of chemical compounds against the pathological strains based on well diffusion assay.

Chem. compd.	Gram-positive bacteria				Gram-negative bacteria			Yeast	
	*Staphylococcus aureus ATCC29213*	*B. subtilis ATCC6633*	*B. megaterium ATCC9885*	*Sarcina lutea *	*Klebsiella pneumoniae ATCC13883*	*Pseudomonas aeruginosa ATCC27953*	*E. coli ATCC25922*	*Saccharomyces cerevisiae *	*Candida albicans* NRRL Y-477
**5**	18	17	20	23	24	23	24	22	18
**8a**	19	16	18	18	17	19	23	20	18
**8b**	19	18	17	28	18	19	24	21	18
**8c**	25	26	23	30	34	33	31	22	23
**8d**	23	12	17	18	16	18	24	20	18
**13a**	22	18	28	21	23	19	23	20	16
**13b**	20	18	19	22	20	21	24	19	16
**14**	19	18	20	23	22	22	25	24	20
**15**	20	17	20	24	21	19	25	20	18
**16**	19	16	21	20	21	20	27	21	16
Ciprofloxacin	20	22	24	20	25	24	23	N.A.	N.A.
Ketoconazole	N.A.	N.A.	N.A.	N.A.	N.A.	N.A.	N.A.	23	22

The experiment was carried out in triplicate, and the average zone of inhibition was calculated; N.A. (no activity).

**Table 3:** Minimum inhibitory concentration (*μ*g/mL) against the pathological strains based on twofold serial dilution technique.

Chem. compds	Gram-positive bacteria				Gram-negative bacteria			Yeast	
	*Staphylococcus aureus ATCC29213*	*B. subtilis ATCC6633*	*B. megaterium ATCC9885*	*Sarcina lutea*	*Klebsiella pneumoniae ATCC13883*	*Pseudomonas aeruginosa ATCC27953*	*E. coli ATCC25922*	*Saccharomyces* *cerevisiae *	*Candida albicans* NRRL Y-477
**5**	200	—	200	100	100	100	100	100	200
**8a**	19	16	200	100	—	200	100	200	200
**8b**	100	200	—	50	200	200	100	200	—
**8c**	50	50	100	50	25	50	25	200	100
**8d**	50	—	—	100	—	200	100	200	200
**13a**	200	200	200	100	200	200	100	200	—
**13b**	100	200	50	100	100	200	100	200	—
**14**	200	200	200	100	100	100	50	100	200
**15**	200	—	200	10 0	100	200	50	200	200
**16**	200	—	20	100	100	200	50	200	—
Ciprofloxacin	25	25	25	25	25	25	25	N.A.	N.A.
Ketoconazole	N.A.	N.A.	N.A.	N.A.	N.A.	N.A.	N.A.	25	25
